# Relationship Between Oxidative Stress Markers and Endothelin-1 Levels in Newborns of Different Gestational Ages

**DOI:** 10.3389/fped.2020.00279

**Published:** 2020-06-02

**Authors:** Gospodin Stefanov, Seema Briyal, Gwendolyn Pais, Bhagya Puppala, Anil Gulati

**Affiliations:** ^1^Division of Neonatology, Advocate Children's Hospital, Park Ridge, IL, United States; ^2^Chicago College of Pharmacy, Midwestern University, Downers Grove, IL, United States

**Keywords:** oxidative stress, endothelin-1, neonate, glutathione, malondialdehyde, cord blood, newborn

## Abstract

Oxidative stress results from excessive reactive oxygen species formation and/or inadequate antioxidant defense. Premature and critically ill infants are especially susceptible due to an immature intrinsic antioxidant system that cannot fully compensate for a free radical load. Oxidative stress is also associated with endothelial dysfunction and alterations in Endothelin-1 (ET-1) signaling pathways. However, the effects of the complex interaction between oxidative stress and ET-1 in newborns are not well-understood. The objective of this pilot study was to determine the relationship between levels of common oxidative stress biomarkers [glutathione (GSH), malondialdehyde (MDA)] and ET-1 in newborns of different gestational ages. In a level IV NICU, 63 neonates were prospectively enrolled and divided into groups based on gestational age at birth: Early Preterm (24 0/7–30 6/7 weeks), Late Preterm (31 0/7–36 6/7 weeks), and Term (37 0/7–42 weeks). Umbilical cord (1.5 mL) and 24(±4) h of life (24 h) (1 mL) blood samples were collected for GSH, MDA, and ET-1 analyses. GSH, MDA, and ET-1 were determined using established methodology. Mean cord MDA levels for all age groups, Early Preterm (2.93 ± 0.08 pg/ml), Late Preterm (2.73 ± 0.15 pg/ml), and Term (2.92 ± 0.13 pg/ml), were significantly higher than those at 24 h of life (*p* < 0.001). Mean cord ET-1 levels were significantly higher than 24 h samples in both Early and Late Preterm groups (*p* < 0.05). Cord and 24 h ET-1 levels did not correlate with MDA and GSH levels at birth (r^2^ = 0.03, *p* > 0.05 and r^2^ = 0.001, *p* > 0.05, respectively) or 24 h of life (r^2^ = 0.001, *p* > 0.05 and r^2^ = 0.03, *p* > 0.05, respectively). Preterm neonates exposed to prenatal corticosteroids (1.87 ± 0.31 pg/ml) had lower cord MDA levels than non-exposed neonates (2.85 ± 0.12 pg/ml) (*p* < 0.05). Both cord and 24 h OS markers were significantly higher in neonates treated with oxygen therapy (*p* < 0.005 and *p* < 0.05, respectively) than those who did not receive supplemental oxygen. Oxidative stress markers (MDA and GSH) and ET-1 levels act independently. MDA is higher in cord blood than at 24 h of life regardless of gestational age. In preterm neonates, ET-1 levels are higher in umbilical cord blood compared to 24 h of life.

## Introduction

Redox homeostasis relies on the critical interplay between reactive oxygen species (ROS) production and the ability of the body's antioxidant defense system to subsequently inactivate it. Under physiological conditions, generated ROS (made up of free radicals and non-radical forms) activate redox-sensitive transcriptions, ion channels, and enzymes (e.g., protein kinases) (1). Excessive free radicals are then neutralized by antioxidants that are either endogenous/enzymatic (e.g., superoxide dismutase, catalase, and glutathione) or exogenous/non-enzymatic (e.g., vitamins A, C, E, and selenium) (2, 3). However, alterations in this fragile balance—known as oxidative stress—are caused by excessive ROS formation, impaired ROS inactivation due to an overwhelmed intrinsic antioxidant defense, or a combination of both. Oxidative stress (OS) results in damage to lipids, proteins, and nucleic acids followed by cellular injury and even cell death (4, 5).

In the neonate, redox homeostasis is burdened at birth during the transition to extrauterine life which involves a series of physiological changes that significantly increase both ROS production and the potential for OS (6, 7). While mature and healthy infants are able to adapt to these changes, preterm, and sick neonates are at a greater risk for OS-related injury due to immature endogenous and insufficient exogenous antioxidant protection (8, 9). In addition, the threat of OS injury in the preterm infant is exacerbated if perinatal conditions (e.g., preeclampsia, hypoxia, and respiratory distress) or treatments (e.g., oxygen therapy) reduce their antioxidant capacity and increase ROS production further (10, 11). ROS have been implicated in the pathogenesis of many neonatal morbidities, such as retinopathy of prematurity, hypoxic-ischemic brain injury, intraventricular hemorrhage, and chronic lung disease (10, 12).

Interestingly, OS has also been shown to be associated with endothelial dysfunction in cardiovascular disease and alterations in both ET-1 and nitric oxide (NO) signaling pathways (13, 14). As a potent vasoconstrictor, ET-1 plays a key role in vascular homeostasis. The effects of the complex interaction between OS and ET-1 on neonatal cardio-respiratory adaptation and morbidities are not well-understood.

Therefore, the purpose of this pilot study was to determine the relationship between oxidative stress markers and ET-1 levels during the early neonatal period in newborns of different gestational ages who were admitted to the NICU.

## Materials and Methods

### Study Population

Sixty-three subjects were enrolled into this prospective, observational pilot study that was conducted over a 21-month period in a Level IV NICU. All study procedures were approved by the local Institutional Review Board and performed only after written parent permission was obtained. Inclusion criteria required both inborn status and a gestational age (GA) at birth between 24 0/7 and 42 0/7 weeks. Infants were ineligible if they presented with a congenital malformation that was incompatible with life or any other condition that would compromise the infant's safety, in the opinion of the investigator.

Following enrollment, subjects were divided into groups dependent upon their birth GA in order to discern differences related to GA: Group 1—Early Preterm (24 0/7–30 6/7 weeks), Group 2—Late Preterm (31 0/7–36 6/7 weeks), and Group 3—Term (37 0/7–42 weeks). Data was collected on all enrolled subjects through their first week of life. Table 1 depicts maternal and infant demographic data analyses by group.

**Table 1:** Demographics by age group.

	**Group 1: early preterm**	**Group 2: late preterm**	**Group 3: term**
	**(24 0/7–30 6/7 weeks)**	**(31 0/7–36 6/7 weeks)**	**(37 0/7–42 weeks)**
	**(*n* = 24)**	**(*n* = 26)**	**(*n* = 13)**
**MATERNAL DATA**			
Age (yrs.)	28.9 ± 5.43^*^	31.1 ± 4.82^*^	30.1 ± 6.16^*^
Prenatal steroids (*n*) (%)	20 (83.3%)	9 (34.6%)	0 (0%)
**MODE OF DELIVERY** (*n*) (%)			
Vaginal delivery	10 (41.7%)	11 (42.3%)	5 (38.5%)
Cesarean section	14 (58.3%)	15 (57.7%)	8 (61.5%)
**APGAR SCORES**			
1 min	6.4 ± 2.08^*^	7.8 ± 1.54^*^	7.3 ± 1.55^*^
5 min	8.2 ± 1.27^*^	8.9 ± 0.33^*^	8.6 ± 0.65^*^
**MATURITY (GA) (wks.)**			
Gestational age	28.8 ± 2.17^*^	33.8 ± 1.28^*^	38.4 ± 1.57^*^
**WEIGHT (g)**			
Birth weight	1194.8 ± 369.65^*^	2175 ± 412.73^*^	2971.5 ± 397.73^*^
**INTRAUTERINE GROWTH (*****n*****) (%)**			
Small for GA	6 (25%)	4 (15.4%)	5 (38.5%)
Large for GA	0 (0%)	2 (7.7%)	0 (0%)
**GENDER (*****n*****) (%)**			
Female	9 (37.5%)	11 (42.3%)	3 (23.1%)
Male	15 (62.5%)	15 (57.7%)	10 (76.9%)
**ETHNICITY (*****n*****) (%)**			
African American (non-Hispanic)	3 (12.5%)	1 (3.8%)	0 (%)
Asian	1 (4.2%)	3 (11.6)	3 (23.1%)
Caucasian	13 (54.1%)	18 (69.2%)	9 (69.2%)
Hispanic	7 (29.2%)	4 (15.4%)	1 (7.7%)

* *Mean ± SD*.

### Blood Sample Collection for Oxidative Stress Markers and ET-1 Analyses

Under aseptic conditions, 1.5 mL of umbilical cord blood was collected in EDTA-containing tubes at the time of delivery. At 24 (±4) hours (h) of life, an additional 1 mL blood sample was drawn from an indwelling catheter or heel stick. The samples were immediately placed on ice and/or refrigerated. Within 4 h of collection, all specimens were processed and frozen at −70°C to ensure stability until laboratory analyses of ET-1 and OS markers [reduced glutathione (GSH) and malondialdehyde (MDA)] were performed at Midwestern University, Chicago College of Pharmacy, Downers Grove, IL.

Additional laboratory results (i.e., bilirubin at 12 h and/or 24 h of life) analyzed in this study were performed solely as a part of the neonates' standard of care. These additional levels were only included in the analyses if drawn within ±4 h of the 24 h-of-life research sample.

### Estimation of Oxidative Stress Marker Levels

#### Lipid Peroxidation Measurement

MDA, the indicator of lipid peroxidation, was estimated according to the method of Okhawa et al. (15). The reagents: acetic acid [1.5 mL (20%) pH 3.5], thiobarbituric acid [1.5 mL (0.8%)], and sodium dodecyl sulfate [0.2 mL (8.1%)], were added to 0.1 mL of processed blood sample. The mixture was then heated at 100°C for 60 min. The mixture was cooled with tap water and 5 mL of n-butanol: pyridine (15:1% v/v) and 1 mL of distilled water, was added. This mixture was shaken vigorously. After centrifugation at 4,000 rpm for 10 min, the organic layer was withdrawn and absorbance was measured using a spectrophotometer at 532 nm.

#### Glutathione Measurement

Measurement of Glutathione: GSH was measured according to the method of Ellman (16) with minor modification. Briefly, the sample was centrifuged with 5% trichloroacetic acid to centrifuge out the proteins. To 0.1 mL of this homogenate, 2 mL of phosphate buffer (pH 8.4), 0.5 mL of 5′5 dithiobis (2-nitrobenzoic acid) (DTNB) and 0.4 mL of double-distilled water was added. The mixture was vortexed and the absorbance read at 412 nm within 15 min using a spectrophotometer.

### Estimation of ET-1 Levels

Plasma ET-1 concentrations were estimated in duplicate using a commercially available enzyme immunoassay kit (Enzo Life Sciences, Farmingdale, NY) (17) following the manufacture's protocol. Briefly, plasma samples and standards were added to wells coated with a monoclonal antibody specific for ET-1. The plate was then washed after 24 h of incubation and horseradish peroxidase (HRP) labeled monoclonal antibody was then added. After 30 min of incubation the plate was washed and a solution of 3,3_,5,5_-tetramethylbenzidine substrate was added which generates a blue color. Hydrochloric acid (1N) was added to stop the substrate reaction and the resulting yellow color was read at 450 nm using DTX 800 Multimode detector and the data was analyzed with Multimode Detection Software (Beckman Coulter, Inc., Harbor Boulevard, Fullerton, CA). The measured optical density is directly proportional to the concentration of ET-1.

### Statistical Analyses

All data are reported as mean ± SD and mean ± SEM. Evaluation of the relationships between ET-1, OS markers and OS-related factors was calculated using Pearson's coefficient of correlation test. Significance between groups was determined using student-*t* test and Two-way analysis of variance test (ANOVA) followed by Tukey's multiple comparisons test. A *p* < 0.05 was considered as statistically significant. Statistical analyses were performed using GraphPad Prism 8.0 (San Diego, CA, USA).

## Results

### Umbilical Cord and 24 h Oxidative Stress Markers by GA

**Figure 1:**
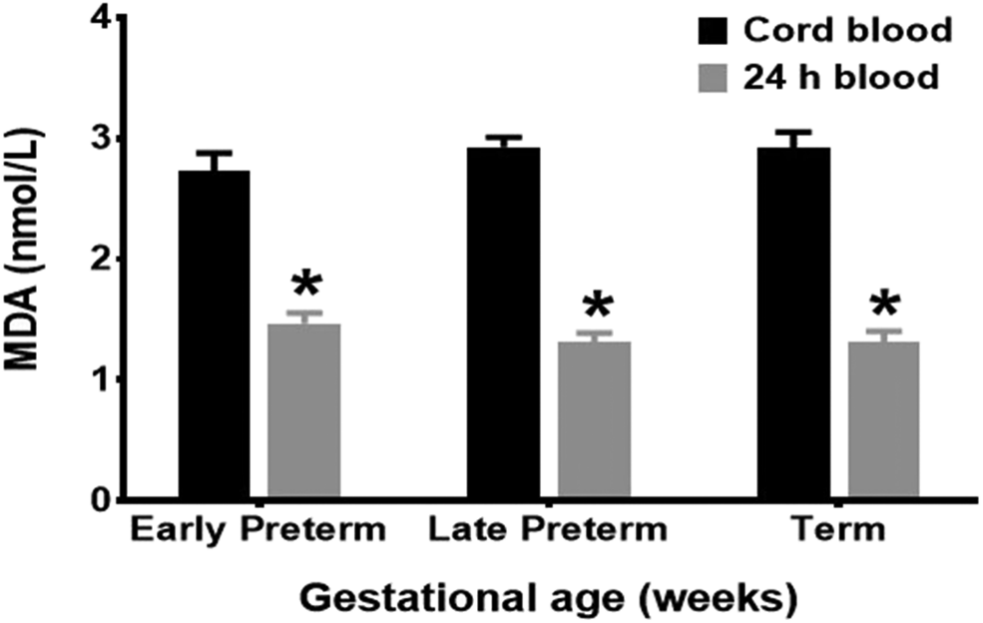
MDA levels by gestational age groups. Mean cord MDA levels, Early Preterm (2.93 ± 0.08 nmol/L), Late Preterm (2.73 ± 0.15 nmol/L), and Term (2.92 ± 0.13 nmol/L), were significantly higher than those obtained at 24 h of life (*p* < 0.001).

Mean cord MDA levels for all age groups, Early Preterm (2.93 ± 0.08 nmol/L), Late Preterm (2.73 ± 0.15 nmol/L), and Term (2.92 ± 0.13 nmol/L), were significantly higher than those obtained at 24 h of life (*p* < 0.001) (Figure 1).

Mean GSH levels did not differ significantly (*p* > 0.05) between umbilical cord and 24 h samples among any of the three GA groups.

### Umbilical Cord and 24 h of Life ET-1 Levels by GA

The mean umbilical cord ET-1 levels were significantly higher (*p* < 0.05) than 24 h samples in both the Early and Late Preterm groups. No significant difference (*p* > 0.05) was found, between mean ET-1 umbilical cord and 24 h levels in the Term group (Table 2).

**Table 2:** Umbilical cord and 24 h of Life ET-1 levels by GA.

**Specimen type**	**Groups**	**Umbilical cord**	**24-h of life**	***p*-value**
Plasma ET-1 (pg/mL)	Preterm 1 24–30 6/7 wks (*n* = 24)	7.57 ± 0.82^*^ (1.58–14.25)∧	4.90 ± 0.71^*^ (0.14–14.83)∧	0.0146
	Preterm 2 •31–36 6/7 wks (*n* = 26)	5.77 ± 0.70^*^ (0.14–13.89)∧	3.82 ± 0.51^*^ (0.14–8.94)∧	0.0289
	Full term •37–42 wks (*n* = 13)	7.67 ± 1.02^*^ (0.14–12.88)∧	5.99 ± 0.98^*^ (1.67–12.97)∧	0.247

* *Mean ± SEM, ^∧^Range*.

### Umbilical Cord and 24 h Oxidative Stress Markers

Only a weak negative correlation was found between the two OS markers, MDA and GSH, in cord blood (r^2^ = 0.025, *p* > 0.05 and r^2^ = 0.0005, *p* > 0.05, respectively) and 24 h samples (r^2^ = 0.0007, *p* > 0.05 and r^2^ = 0.032, *p* > 0.05, respectively).

### ET-1 Levels and Oxidative Stress Markers by GA

Cord and 24 h ET-1 levels did not correlate with MDA and GSH levels at birth (r^2^ = 0.03, *p* > 0.05 and r^2^ = 0.001, *p* > 0.05, respectively) or at 24 h of life (r^2^ = 0.001, *p* > 0.05 and r^2^ = 0.03, *p* > 0.05, respectively).

### ET-1 Levels and Oxidative Stress Markers by Size-at-Birth Subgroups

Analysis of size differences in both the early and late preterm groups demonstrated no significant difference (*p* > 0.05) between umbilical cord and 24 h levels of ET-1, MDA, or GSH in either the small for gestational age (SGA) or appropriate for gestational age (AGA) subgroups.

### Oxidative Stress Markers

#### Oxidative Stress Markers and Apgar Scores

The 1-min Apgar score did not correlate with MDA or GSH in cord (r^2^ = 0.0006, *p* > 0.05 and r^2^ = 0.0225, *p* > 0.05, respectively) or 24 h blood samples (r^2^ = 0.0001, *p* > 0.05 and r^2^ = 0.01319, *p* > 0.05, respectively). Similarly, no correlation was found between the 5-min Apgar score and MDA and GSH levels in cord (r^2^ = 0.0004, *p* > 0.05 and r^2^ = 0.0081, *p* > 0.05, respectively) or 24 h samples (r^2^ = 0.00085, *p* > 0.05 and r^2^ = 0.0078, *p* > 0.05, respectively).

#### Oxidative Stress Markers and Prenatal Corticosteroids

The mean umbilical cord MDA levels in preterm neonates exposed to prenatal corticosteroids (1.87 ± 0.31 pg/ml) were significantly lower (*p* < 0.05) than those obtained from neonates whose mothers did not receive prenatal corticosteroids (2.85 ± 0.12 pg/ml). However, no significant difference (*p* > 0.05) was found between 24 h MDA levels. GSH levels (both umbilical cord and 24 h) did not differ significantly (*p* > 0.05) between these two subgroups.

#### Oxidative Stress Markers and Oxygen Therapy

Mean MDA and GSH levels were significantly higher in neonates who received oxygen therapy at the time of delivery or thereafter in umbilical cord (*p* < 0.005 and *p* < 0.05, respectively) and 24 h blood samples (*p* < 0.005 and *p* < 0.05, respectively) than those obtained from neonates who were not exposed to supplemental oxygen (Table 3).

**Table 3:** Oxidative stress markers and oxygen therapy.

**OS markers**	**O_**2**_ therapy** **(*n* = 23)**	**No O_**2**_ therapy** **(*n* = 40)**	***p*-value**
Umbilical Cord MDA	2.78 ± 0.14^*^ (1.31–3.58)∧	1.83 ± 0.23^*^ (1.33–3.47)∧	0.0049
24 h MDA	1.42 ± 0.08^*^ (0.89–1.67)∧	0.84 ± 0.12^*^ (0.59–2.20)∧	0.0013
Umbilical Cord GSH	0.62 ± 0.07^*^ (0.49–0.94)	0.36 ± 0.07^*^ (0.47–1.19)	0.0221
24 h GSH	0.61 ± 0.08^*^ (0.45–1.12)∧	0.37 ± 0.06^*^ (0.45–0.94)∧	0.0227

* *Mean ± SEM, ^∧^Range*.

#### Oxidative Stress Markers and Mode of Delivery, Gender and Race/Ethnicity

The mode of delivery (vaginal or cesarean section) or gender of the neonates did not affect a significant difference (*p* > 0.05) between OS markers levels in umbilical cord (*p* > 0.05) or 24 h samples. Similarly, no significant differences (*p* > 0.05) were found when analyzing neonatal race/ethnicity with MDA or GSH levels in umbilical cord or 24 h-blood samples.

## Discussion

The changes in equilibrium between generated pro- and antioxidants that result in ROS lead to oxidative stress. Multiple factors, such as hyperoxia, hyperglycemia, hypoxia, enhance free radical production (18). Newborns are especially prone to OS-related injury due to an immature antioxidant defense and exposure to high oxygen concentrations, infections, and perinatal distress (9, 19). Preterm and low birth weight newborns have a significantly reduced antioxidant capacity that may predispose them to an OS-related injury (10). Recently, both *in-vitro* and *in-vivo* specific biomarkers for OS have been introduced into the field of pediatrics (5, 20).

Black et al. suggested OS could be associated with alterations in the ET-1 signaling pathways, and conversely, ET-1-induced vasoconstriction may be dependent on the production of superoxide anion (21). ET-1 is a potent vasoconstrictor peptide involved in the development of persistent pulmonary hypertension, hypoxic brain injury, and bronchopulmonary dysplagia (22). A number of studies have implicated oxidative stress in the development of endothelial dysfunction and the pathogenesis of cardiovascular disease. Furthermore, oxidative stress has been shown to be associated with alterations in both the ET-1 and nitric oxide (NO) signaling pathways. Reactive oxygen species (ROS) generated in oxidative stress is known to regulate cellular level of ET-1 and facilitates its secretion (13, 14). Oxidative stress is also known to decrease the bioavailable NO which leads to potentiate ET-1 signaling. This evidence suggests the link between ET-1 and oxidative stress. The present study found no correlation between ET-1 and two important OS biomarkers (MDA and GSH) in cord and 24 h blood samples. These findings could be explained by the complexity of the transitional perinatal period that may reflect a mixed maternal and neonatal redox status (23).

The results of our study demonstrated that ET-1 levels were higher in umbilical cord blood compared to 24 h-of-life samples in both Early and Late preterm groups. ET-1 is known to contribute to placental vasoconstriction and could enhance ROS production. Recent studies reported that maternal endothelial dysfunction may be related to OS and could impact fetal development and outcome (24).

MDA is an OS biomarker extensively used to detect lipid peroxidation. Data from our study has demonstrated that MDA levels in all neonatal age groups were significantly higher in cord blood than those obtained at 24 h of life (25, 26). Our results indicate that during the period of transition to extrauterine life, lipid peroxidation in neonates surges at the time of delivery with a subsequent decline to lower levels compared to GSH levels that reflect mostly DNA and protein antioxidant defense (27, 28).

Oxygen as a highly reactive species is a potent trigger of free radical production (29). Our results showed that OS marker levels were significantly higher in neonates that received oxygen therapy at or after delivery compared to neonates who were not administered supplemental oxygen. Oxygen therapy could be one of the factors increasing OS marker levels in neonates.

Hypoxia, paradoxically, can also increase the formation of free radicals (i.e., superoxide anion) (2). Sridhar et al. reported that 1- and 5-min Apgar scores are reliant on cord MDA levels in neonates with intrauterine growth restriction (30). However, Abessolo et al. found no correlation between antioxidant enzymes (i.e., superoxide dismutase, glutathione peroxidase) and the 5-min Apgar score (31). No significant correlation was found between both OS markers (MDA and GSH) and Apgar scores in our study.

Our findings also demonstrated that umbilical cord MDA levels of preterm neonates exposed to prenatal corticosteroids were significantly lower than those obtained from preterm neonates whose mothers did not receive it. Crowther et al. have shown that exposure to prenatal corticosteroids reduces respiratory distress syndrome and oxygen therapy requirements of neonates post-delivery (32). Therefore, prenatal steroids could interfere with redox homeostasis directly via the lipid peroxidation pathway and/or indirectly by affecting neonatal morbidities, such as respiratory distress syndrome (33).

Recent studies have reported that an elevated maternal OS marker—protein carbonyls—is associated with a lower birth weight, smaller head circumference, and an increased risk of reduced fetal growth (delivery of an SGA newborn). Evidence has also been published that markers of lipid peroxidation (but not proteins or DNA) were significantly higher in an intrauterine growth retardation group of patients (34, 35). Our study found no significant difference between levels of OS markers in SGA and AGA preterm groups. Pathophysiological causes of intrauterine growth restriction are complex including, maternal nutrition and chronic diseases, preeclampsia, various fetal and placental determinants affecting uteroplacental and fetal blood flow (36).

## Limitations

A limitation of our study was an inability to evaluate a possible correlation between OS markers and hyperglycemia. This was due to a large variation in draw times of blood glucose samples obtained as a part of the neonates' standard of care. Only a small number of glucose levels were drawn within ±4 h period of OS marker sampling.

## Conclusion

Oxidative stress marker malondialdehyde (MDA) is higher in cord blood than at 24 h of life regardless of gestational age. In preterm neonates, ET-1 levels are higher in umbilical cord blood compared to 24 h of life.

## Data Availability Statement

The datasets generated for this study are available on request to the corresponding author.

## Ethics Statement

The studies involving human participants were reviewed and approved by Advocate Health Care Institutional Review Board. Written informed consent to participate in this study was provided by the participants' legal guardian/next of kin.

## Author Contributions

GS, BP, and AG contributed to study conception design and methodology. GS developed the database, obtained consent, collected samples, performed all data collection, and completed the first draft of the manuscript. SB and GP performed biomarker estimations. GS, SB, and GP all contributed to statistical analyses. GS, SB, GP, BP, and AG contributed to data analysis and critical review of the manuscript. GS, SB, GP, BP, and AG read and approved the submitted manuscript version.

## Conflict of Interest

AG is founder and CEO of company Pharmazz Inc. The remaining authors declare that the research was conducted in the absence of any commercial or financial relationships that could be construed as a potential conflict of interest.

## Abbreviations

AGA: Appropriate for gestational age
ET-1: Endothelin−1
GA: Gestational age
GSH: Glutathione
h: Hours
MDA: Malondialdehyde
NICU: Neonatal intensive care unit
NO: Nitric oxide
OS: Oxidative stress
ROS: Reactive oxygen species
SGA: Small for gestational age.
